# Clinical significance of serum lipids in idiopathic pulmonary alveolar proteinosis

**DOI:** 10.1186/1476-511X-11-12

**Published:** 2012-01-17

**Authors:** Cun S Fang, Ying C Wang, Tao H Zhang, Jing Wu, Wei Wang, Chun Wang, Ming Y Zhang

**Affiliations:** 1 Nine department of Respiratory Medicine, Nanjing Chest Hospital, 215 Guangzhou Road, Nanjing, 210029, P.R. China

**Keywords:** pulmonary alveolar proteinosis, high-density lipoprotein cholesterol lipid, low-density lipoprotein cholesterol, whole lung lavage

## Abstract

**Background:**

It is well known that pulmonary alveolar proteinosis(PAP) is characterised by accumulation of surfactant lipids and proteins within airspaces. However, few previous data describe the serum lipid levels associated with PAP.

**Materials and methods:**

We retrospectively reviewed 25 patients with idiopathic PAP(iPAP). The serum lipid levels of patients with idiopathic PAP were compared with those of the healthy volunteers. In patients and healthy subjects, the LDL-C/HDL-C ratios were 2.94 ± 1.21 and 1.60 ± 0.70, respectively (*p *< 0.001), HDL-C were 1.11 ± 0.27 and 1.71 ± 0.71 respectively (*p *< 0.001). The values of LDL-C correlated significantly with those of PaO2 and P_A-a_O2 (r = -0.685, *p *= 0.003, and r = 0.688, *p *= 0.003, respectively). The values of LDL-C/HDL-C ratios also correlated with PaO2 levels and PA-aO2 levels (r = -0.698, p = 0.003, and r = 0.653, p = 0.006, respectively). 11 and 13 patients experienced respectively a decline in TC and LDL-C levels following whole lung lavage(WLL), the median decline was 0.71 mmol/L(*p *< 0.009) and 0.47 mmol/L(*p *< 0.003), respectively.

**Conclusions:**

the serum lipid levels, especially the levels of LDL-C and LDL-C/HDL-C, may reflect the severity of the disease in PAP patients, and predict the therapeutic effect of WLL.

## Background

Pulmonary alveolar proteinosis(PAP) is a rare disease characterized by the accumulation of lipoproteinaceous material in the alveoli[1]. Clinically, Three forms of PAP have been described: congenital, secondary, and idiopathic. More than 90% of patients are idiopathic PAP(iPAP), is specifically associated with the presence of granulocyte-macrophage colony stimulating factor(GM-CSF) autoantibodies that are thought to mediate pathogenesis by eliminating GM-CSF bioactivity, thereby this loss of functional GM-CSF results in a filling of the alveolar spaces of the lungs with the lipoproteinaceous material called pulmonary surfactant[2,3].

Pulmonary surfactant is comprised of 90% lipid, 10% protein, and less than 1% carbohydrate. Cholesterol is the major neutral lipid (up to 90%) in pulmonary surfactant. At least 80% of the cholesterol present in the lung[4], and virtually all that in surfactant, is derived from circulating lipoproteins, with very low-density lipoprotein believed to be the major vehicle of delivery to the lung [5]. Therefore, impaired lipid metabolism may play an important role in the development of iPAP.

It has been reported[6] that serum levels of triglyceride were higher in patients with idiopathic PAP, while HDL-C levels were lower in patients. Similarly, serum levels of cholestenoic acid were also significantly increased in the PAP patients[7]. Elevated serum cholesterol levels have been confirmed in 19% of PAP patients[1].

Based on these findings, the clinical relevance of lipid metabolism in iPAP deserves further study. The current study investigated these relationships to determine whether serum lipid levels would provide valuable clinical information to assess and monitor disease progression. We measured serum lipid levels from iPAP patients and investigated their relation to severity markers for iPAP including serum lactate dehydrogenase (LDH), arterial blood gases. We also assessed variations of these lipids before and after whole lung lavage(WLL).

## Materials and methods

### Patients

We retrospectively reviewed 25 patients with idiopathic PAP that were diagnosed by cytological examination of BAL fluid and pathological examination of the lung tissues. The aetiologies associated with secondary PAP were not found in these patients. Data for the iPAP patients were collected from Nanjing Chest Hospital (Bronchalveolar Lavage Center) between 2006 and 2011. Patients were excluded from the study if they had a history of diabetes mellitus, chronic liver or kidney disease, cancer, or use of corticosteroids or lipid-lowing drugs.

### Control groups

Using a case-controlled study design, data from healthy volunteers(HA) were obtained from persons who were examined in the Nanjing Chest Hospital between April 2010 and May 2011. All healthy volunteers were free of symptoms and not taking any medications. The study protocol was approved by the Human Ethics Review Committees of Nanjing Chest Hospital and all study participants provided written informed consent.

### Assays

Pulmonary function testing(YLS9-HI-101, Japan) including spirometry, plethysmography, carbon monoxide lung transfer factor (TLCO), and analyses of arterial blood gases(BJ05-400, Germany) were performed in most of patients. Blood samples for PaO2 and PaCO2 values were analysed at room air. PAO2 (alveolar oxygen tension) is calculated by the following equation. PAO2 = (barometric pressure - 47) × FiO2 -PaCO2/R. R, an exchange ratio, is assumed as 0.8 in this study. The alveolar arterial PO2 difference (P_A-a_O2) is calculated by subtracting PaO2 from PAO2. Laboratory examinations were performed on blood samples obtained after an overnight fasting. The levels of serum lipid levels (total cholesterol (TC), triglyceride (TG), low-density lipoprotein cholesterol (LDL-C), high-density lipoprotein cholesterol (HDL-C)), apolipoprotein-A1 (Apo-A1) and apolipoprotein-B (Apo-B), Lipoprotein(a)), and lactate dehydrogenase (LDH) were measured with commercial kits using an automated chemistry analyzer (OI Analytical, American).

### Criteria of WLL

Whole lung lavage, the current standard of care for treating autoimmune PAP, is effective in physically removing the accumulated surfactant and is effective in most patients[8]. The criteria for therapeutic lung lavage in this study were as follow[9]: (1) presence of persistent or progressive respiratory failure; (2) absence of respiratory difficulty at rest, but presence of exercise desaturation; (3) a significant limitation in daily or sport activities. Patients were examined with lipid levels and arterial blood gas analysis after one week of WLL.

### Statistical analysis

Statistics were performed with SPSS version 11.0. Paired data comparisons were performed using a Wilcoxon signed rank test. The nonparametric Mann-Whitney U-test was used when data were not normally distributed. The correlations between variables were determined by Spearman rank correlation coefficients. P values of less than 0.05 were considered statistically significant.

## Results

It is indicated that the median age at diagnosis was 41 years(table 1). 22 of 25 patients are men, and 76% have a history of smoking. Nine patients had a occupational history of contact with dust, metal, silica, etc. the duration of occupational exposure ranged from 2 to 17 years. Most patients had comorbidity: pneumonia, tuberculosis, and fatty liver were the most common. 7 of 25 patients(28%) suffered from varying degrees of fatty liver diagnosed by B Type Ultrasonography. On pulmonary function testing, the most common pattern seen is that of a restrictive defect, with a disproportionate reduction in diffusing capacity. Arterial blood gas analysis showed hypoxemia with 41% pressure of oxygen (PaO2) below 60 mmHg, While arterial carbon dioxide pressure(PaCO2) was not significantly changed.

**Table 1:** Baseline characteristics of patients

Characteristics	N	mean ± SD
Gender (M/F)	22/3	
Age(years)	25	41 ± 8.7
Smoking	19	
Dust exposure	9	
**Pulmonary function**		
FEV1(L)	21	2.73 ± 0.5
FEV1/FVC(%)	21	88.89 ± 5.47
TLC (L)	21	4.03 ± 1.06
DLCO(mmol/min/kpa)	21	4.66 ± 1.82
**Blood gas analysis**		
PaO2 (mmHg)	17	65 ± 14
PaCO2 (mmHg)	17	39 ± 3
PA-aO2 (mmHg)	17	36 ± 15
**Serology**		
TC(mmol/L)	25	5.01 ± 1.08
TG(mmol/L)	25	1.7 ± 1.06
HDL-C(mmol/L)	25	1.11 ± 0.27
LDL-C(mmol/L)	25	3.07 ± 1.07
Apo-A1(mmol/L)	25	1.16 ± 0.38
Apo-B(mmol/L)	25	0.94 ± 0.28
Lipoprotein(a) (mg/L)	25	172 ± 63.6
LDH(U/L)	25	239 ± 78
**Comorbidity**		
Pneumonia	5	
Tuberculosis	4	
Fatty Liver	7	

**Figure 1:**
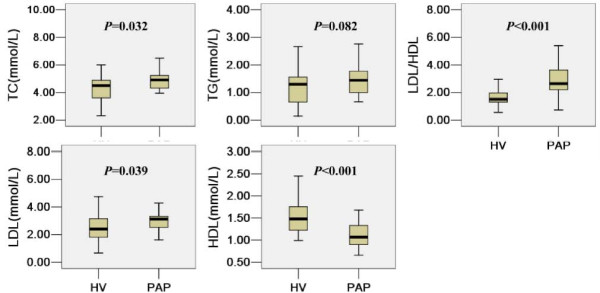
**Comparison of lipid levels between iPAP and HA**.

The serum lipid levels of patients with iPAP were compared with those of the healthy volunteers(HA), as shown in Figure 1. Except for HDL-C, patients with iPAP had higher levels of TC, TG, LDL-C, LDL-C/HDL-C, and lipoprotein(a) than did healthy controls. However, there were no significant differences in TG between the two groups(*p *> 0.05). In patients and healthy subjects, the LDL-C/HDL-C ratios were 2.94 ± 1.21 and 1.60 ± 0.70, respectively (*p *< 0.001), HDL-C were 1.11 ± 0.27 and 1.71 ± 0.71 respectively (*p *< 0.001), LDL-C were 3.07 ± 1.07 and 2.43 ± 0.95 respectively(*p *< 0.05), and Lipoprotein(a) were 172 ± 64 and 153 ± 85 respectively (*p *< 0.05).

Clinically, serum LDH level, PaO2, and alveolararterial P_A-a_O2 have commonly been used to indicate the severity of iPAP[1,10]. The relations between these severity markers and serum lipid levels were analyzed, and the results are summarized in table 2. The values of LDL-C correlated significantly with those of PaO2 and P_A-a_O2 (r = -0.685, *p *= 0.003, and r = 0.688, *p *= 0.003, respectively). A similar pattern was seen with LDL-C/HDL-C ratios correlated negatively with PaO2 levels (r = -0.698, *p *= 0.003) and positively with P_A-a_O2 (r = 0.653, *p *= 0.006). Apo-A1 was highly correlated with LDH and PaO2, and lipoprotein(a) was highly correlated with PaO2(table 2), whereas levels of TC, TG, HDL-C and Apo-B did not show a correlation with any of the severity markers.

**Table 2:** Relation between lipid levels and severity markers in patients with iPAP

	LDH		PaO2		P(A-a)O2	
	Correlation coefficient	P value	Correlation coefficient	P value	Correlation coefficient	P value
**TC**	-0.264	0.213	-0.222	0.427	0.334	0.223
**TG**	-0.152	0.468	0.297	0.264	-0.262	0.327
**HDL-C**	-0.270	0.192	0.496	0.051	-0.452	0.079
**LDL-C**	0.153	0.465	-0.685	0.003	0.688	0.003
**LDL-C/HDL-C**	0.197	0.345	-0.698	0.003	0.653	0.006
**apoA1**	-0.535	0.006	0.514	0.042	-0.370	0.159
**apoB**	0.099	0.637	-0.041	0.880	0.056	0.837
**Lipoprotein(a)**	0.367	0.078	-0.546	0.016	0.441	0.058

**Figure 2:**
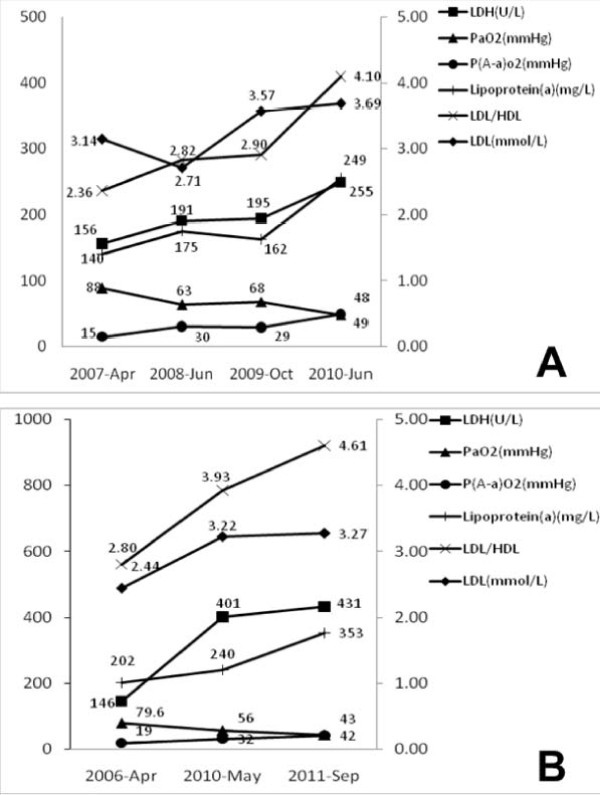
**Serial data of lipid level and severity markers in two iPAP patients with a different clinical course**: (A) a patient who progressivesly deteriorated and (B) a patient who required three cycles of therapeutic lung lavage. In (B), all the data were obtained before each cycle of therapeutic lung lavage.

Serial changes in lipid levels and severity markers for iPAP in two patients are shown in Figure 2. Two patients had a different clinical course. In the patient whose condition rapidly worsened (Figure 2A), the aggravations in serum LDH, PA-aO2 and PaO2 were associated with increase of LDL-C, LDL-C/HDL-C and lipoprotein(a), although LDL-C and lipoprotein(a) were somewhat unparalleled to severity markers at a time. In the patient with three cycles of WLL(Figure 2B), the changes of LDL-C, LDL-C/HDL-C and lipoprotein(a) measured before each cycle of WLL were relatively parallel to severity markers.

**Figure 3:**
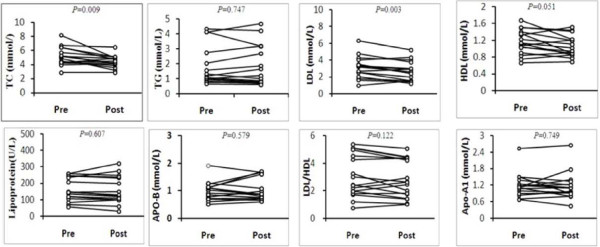
**Paired prelavage and postlavage lipids data from patients with iPAP**.

Whole lung lavage was performed in 19 patients (76%), of whom 2 patients underwent repeated lavage. We analyzed oxygenation and serum lipid levels before and after whole lung lavage(Figure 3). After lavage, substantial improvement is often noted, particularly in dyspnea. hypoxemia was corrected in eighty percent of the patients for whom data were available. P(A-a)O2 and LDH were also improved after lung lavage(additional file 1). serum lipid levels before and after whole lung lavage were available for 16 patients. Eleven patients experienced a decline in TC levels following whole lung lavage, the median decline was 0.71 mmol/L(p < 0.009), A similar pattern was seen with LDL-C in thirteen patients, the median decline was 0.47 mmol/L(*p *< 0.003). LDL-C/HDL-C, TG, HDL-C or lipoprotein(a) showed a slight reduction after lavage, but the difference before and after lavage was not significant.

## Discussion

A growing body of literature suggests that abnormal lipid metabolism was associated with a variety of pulmonary diseases, including chronic obstructive pulmonary disease [11,12]and lung cancer[13,14]. Few previous data describe the lipid profile associated with PAP. This is the first study that has specifically evaluated the clinical value of serum lipid levels in iPAP. We demonstrated that the levels of serum LDL-C and LDL-C/HDL-C were significantly correlated with those of serum PaO2 and P(A-a)O2. These findings indicate that the serum lipid levels, especially the levels of LDL-C and LDL-C/HDL-C, may reflect the severity of the disease in iPAP patients. Patients with iPAP exhibited elevated lipid levels that were reduced significantly after whole lung lavage. In addition, the present study supports the view that impaired lipid metabolism could be present in iPAP[1,6]. These findings may expand the role of serum lipid levels in iPAP.

Several studies have indicated that circulating lipoprotein levels would be changed in iPAP[1,5,6]. Elevated cholesterol levels have been described in 19% of the PAP patients, however, sample size and characteristics were not available[1]. Tian et al.[6] reported that iPAP associated with high triglyceride and low HDL-C levels in the serum. Our results are in consistent with the above findings, the high serum lipid levels did associate with iPAP. Furthermore, twenty-eight percent of the patients in this study suffered from varying degrees of fatty liver which was resulted from hyperlipemia. It was reported that the serum LDL-C/HDL-C ratios could only reflect the severity of the disease[6]. In addition to LDL-C/HDL-C ratios, LDL-C and lipoprotein(a) could also serve as severity indicators in our present study. Furthermore, whether the disease was improve or worsen, the changes in serum LDL-C, LDL-C/HDL-C and lipoprotein(a) were relatively parallel to those of the severity markers in our study. However, this need large cases to conformed.

The mechanisms responsible for dyslipoproteinemia in iPAP are unknown, several observations could support a link between iPAP pathophysiology and abnormal lipid metabolism. The inflammatory cell most implicated in both processes is the macrophage. Macrophages produce phospholipid transfer protein (PLTP) which is predominately found in the lungs[15]. PLTP are involved in HDL-C metabolism and removing cholesterol from the circulation[16]. Furthmore, PLTP can modulate cholesterol deposition in macrophages through its role on oxidative status inside the cells[17]. Alveolar macrophages in PAP patients or murine models were found to exhibit a reduced ability to degrade the surfactant, impaired cell adhesion, ineffective phagocytosis and bacterial killing[18,19]. These features were similar to those of the immature macrophages which had obvious PLTP deficiency[20]. PLTP may be one of the mechanisms explaining dyslipoproteinemia in iPAP. It is unlikely, however, that PLTP is the only factor involved in lipid abnormalities in iPAP. Current thinking indicates that iPAP is an autoimmune disorder characterized by circulating anti-granulocyte macrophage colony stimulating factor (GM-CSF) antibodies and dysfunction in GM-CSF signaling[21]. GM-CSF, which has been verified to involve in the pathophysiological procedure of PAP, could also regulate serum protein and lipid catabolism[22,23]. Further studies are required to clarify these issues.

We further examined the effect of WLL on serum lipid levels, findings demonstrated that the high lipid levels observed prior to WLL would decline with WLL. The effect of WLL in this study was somewhat counter-intuitive. Local lavage can unexpectedly influence the systemic lipid level. the reasonable explanation may be that surfactant proteins produced locally in the lung leak into the circulation. The alveolocapillary membrane, a barrier, can be partitioning the proteins of the pulmonary epithelial lining fluid, but there is some leakage[24]. A range of surfactant proteins has been shown to leak into the circulation. Moreover, accumulated data have shown alveolocapillary membrane permeability was markedly increased in PAP patients[24-26].

There are several limitations of this study. This is a retrospective and single center study, represents a highly selected group of subjects with very severe PAP, and does not allow determination of mechanism. A prospective and comprehensive study to validate clinical significance of lipid metabolism in iPAP would be required. However, the results could be of clinical relevance since all studied subjects came from the same institute, avoiding the inherent bias caused in multicentre studies. In addition, the cases studied were limited because iPAP is a rare disease. Further studies with larger samples of PAP patients are required.

## Conclusions

In summary, we recommend that serum lipid levels would provide valuable clinical information to assess and monitor disease progression and predict the therapeutic effect of WLL. Furthermore, descending hyperlipemia may have potential value to clinical treatment of iPAP.

## List of Abbreviations

PAP: pulmonary alveolar proteinosis; WLL: whole lung lavage; GM-CSF: granulocyte macrophage colony stimulating factor; LDH: lactate dehydrogenase; TLCO: carbon monoxide lung transfer factor; TC: total cholesterol; TG: triglyceride; LDL-C: low-density lipoprotein cholesterol; HDL-C: high-density lipoprotein cholesterol; Apo-A1: apolipoprotein-A1; Apo-B: apolipoprotein-B.

## Competing interests

The authors declare that they have no competing interests.

## Authors' contributions

FSC and ZYM designed the study and carried out the statistical analysis. FSC wrote the manuscript. All authors carried out data collection. All authors read and approved the final manuscript

## Supplementary Material

Additional file 1 **Paired prelavage and postlavage severity markers from patients with iPAP**. PaO2, P(A-a)O2 and LDH were improved after lung lavage. Click here for file
