# Supplementary figures and images for: Clinical significance of serum lipids in idiopathic pulmonary alveolar proteinosis
Source: Lipids Health Dis. 2012 Jan 17;11:12. doi: 10.1186/1476-511X-11-12 (PMC3271981; doi:10.1186/1476-511X-11-12)

Fig1: Paired prelavage and postlavage severity markers from patients with iPAP


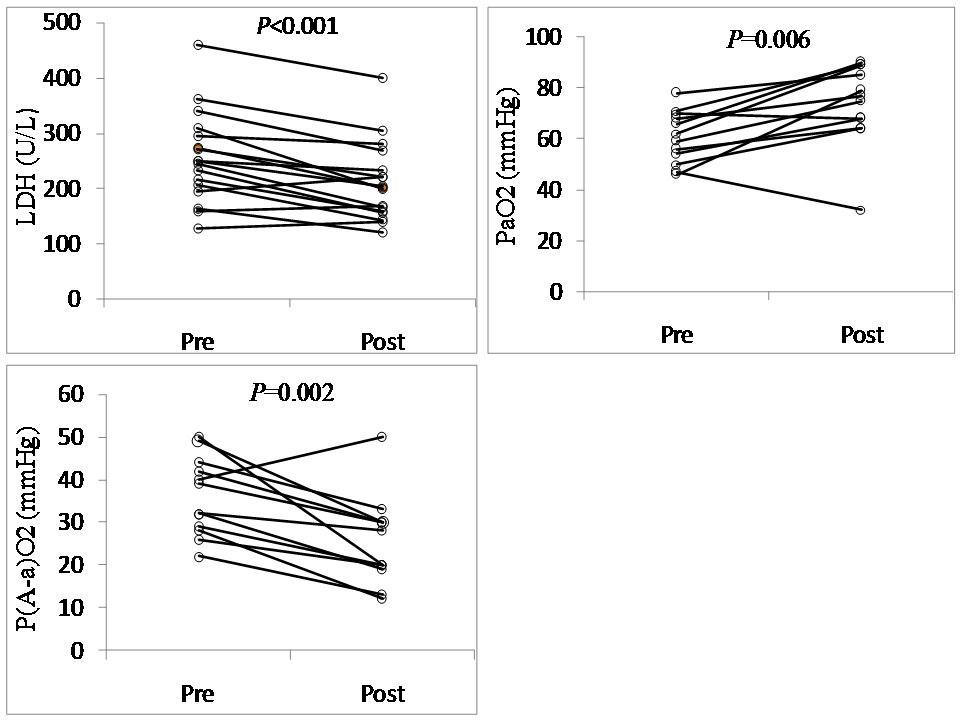

Supplement: Additional file 1 — Paired prelavage and postlavage severity markers from patients with iPAP. PaO2, P(A-a)O2 and LDH were improved after lung lavage. [file 1476-511X-11-12-S1.DOC]
